# Acceleration through Digital Communication: Theorizing on a Perceived Lack of Time

**DOI:** 10.1007/s41463-020-00103-9

**Published:** 2021-01-21

**Authors:** Elisa Maria Entschew

**Affiliations:** grid.461621.6 0000 0001 0728 9327 Dr. Werner Jackstädt Chair of Economic and Business Ethics, HHL Leipzig Graduate School of Management, Leipzig, Germany

**Keywords:** Digital communication, Digitalization, Acceleration, Permanent availability, Time, Rebound effect, Ethics, Moral

## Abstract

Digital communication between humans fundamentally changes the nature of communication. One inherent change is the acceleration of communication as a systematic change in societal life, particularly in the workplace. Often, the aim is to release time resources. However, the acceleration of communication also leads to the opposite: a lack of time. This paradoxical development can be based on an acceleration cycle whereby technologies seem to be a solution on the micro-level, but they are also a significant part of the problem on the meso-level.

## Introduction

Digitalization and globalization have changed human interactions. One significant change relates to communication: humans are communicating digitally more and more. In the course of the Covid-19 crisis, communication via digital media became more present than ever. It promises many beneficial and intended consequences as, for instance, faster communication and time savings. If time is saved, it seems logical to conclude that time becomes abundant. However, time pressure remains in organizations. An alleged solution to this paradoxical development is to implement better technologies to further optimize time. But this would lead to an acceleration cycle resulting in a perceived lack of time.

‘We are living in the most complex and rapid-changing of times. The pace of technological innovation, like never before is challenging the way we operate’ (Martin 2013, p. 33). Surprisingly, this quote is not from the twenty-first century but it is from a nineteenth century edition of the Scientific American journal. Martin refers to this comparison in order to show that acceleration is dependent on the perception of humans. Also, acceleration seems to be relative with regard to the tools available to deal with it: ‘[O]ur tools are way superior to what they have ever been’ (Martin 2013, p. 33).

Hence, tools and technologies enable humans to better deal with acceleration. This is a common claim in the traditional business field promoting the tremendous advantages of digitalization. A widespread assumption of business leaders is that technologies are making humans faster leading to digital ways of doing business by using screens, texts, smartphones and information everywhere (Wajcman 2015). Management reports are often identifying the productivity gains and other advantages through technological acceleration, thereby influencing the mindset of managers (e.g., Chui et al. 2012).

However, the claim neglects an inherent problem: These very technologies reinforce acceleration processes, triggering a self-reinforcing cycle that is not easy to stop. This is due to technologies possibly evoking behavioral responses. On the micro-level, technologies enable humans to be faster and save time. However, on the meso-level the claim falls short, because the usage of new technologies in order to communicate faster, works as a stimulus driving an acceleration cycle leading to the perception of a lack of time (Rosa 2014, pp. 251–252): In the course of everyday work life, organizational members^1^ use their smartphones in order to quickly clarify issues with customers instead of writing a letter. Time is saved. At the same time, work-related email traffic increases continuously resulting in more work which consumes time. This accelerated growth is the result of an organizational process (meso-level) which can hardly be observed by individual organizational members. However, it possibly leads to organizational members perceiving a lack of time.

A general interpretation of this ‘time pressure’ paradox in society can be found in the analysis of Wajcman (2015). Also, the much-respected work of Hartmut Rosa (2014) about social acceleration analyzes acceleration and the ‘time pressure’ paradox in society as a whole referring to transportation, communication, production, private life, etc. While Rosa (2014) identifies the problem of an acceleration cycle, my analysis transfers and extends this theory to the specific field of digital communication in organizations and relates it to the impact of bad management theories and greedy institutions.

The primary contribution of this analysis stems from the dialectic relationship between individual organizational members using communication technologies on the micro-level and the collective organizational process of an acceleration cycle on the meso-level leading to the paradox of a lack of time. Bad management theories (Ghoshal 2005) and greedy institutions (Coser 1974) are critical factors mediating this relationship. In a process of self-fulfilling prophecy, managers are often seen as opportunistic, not trustworthy and rational self-interested maximizers in society (Ghoshal 2005). As a result, the development of greedy institutions is fueled. Greedy institutions tend to have excessive demands on their members. Here, organizations have excessive demands regarding their organizational members’ availability for work-related communication. This paper aims at enriching the traditional business discipline to counteract this negative role of organizations and managers aiming at a heterogenous view of human nature with values that are not only based on self-interest and greed, but also on moral values. After reading the paper, the reader should understand that digital communication leads to the experience of a lack of time if an accelerating communication cycle prevails.

The analysis starts with the clarification of digital communication and acceleration. Subsequently, a theoretical examination of Rosa’s (2014, 2003) acceleration cycle follows. The acceleration cycle is transferred to digital communication, thereby examining the accelerating communication cycle. On this basis, the examples of communicating via letter, email and Slack are analyzed. In this context, possible risks are pointed out. The problem of permanent availability as an attempt to cope with the identified problem on the micro-level is debated in more detail in the last part of this article.

Before jumping into the next section, it is important to note, that the presented acceleration cycle as well as its transfer to digital communication is a (simplifying) model, in the full knowledge that reality is much more complex. Nevertheless, reasons are provided to assume that the described forces prevail in specific settings.

## Digital Communication and Acceleration

The digital revolution of the late twentieth century (which is still ongoing) is concentrated on faster communication and information flows (Augier and Mola 2016, p. 13; Rosa 2014, p. 246). If more and more time is saved through technologies, a logical conclusion would be that organizational members perceive an abundance of time. Still, it seems that organizational members often do not have enough time, despite the fact that they are constantly saving time through acceleration with the use of technological innovations (Rosa 2014, p. 11).

Such perception is reflected in a study on British workplaces. More and more organizational members report they are experiencing work at high speeds and tension (Green 2007). The European Working Conditions Survey also identified this perception. Organizational members report working at very high speed, to tight deadlines or not having enough time to do the job (Parent-Thirion et al. 2017; Parent-Thirion et al. 2012). A possible explanation for this paradox is that activities like communication have risen at a higher rate than the rate of time savings. This encourages the perception of not having enough time. If more and more activities need to be completed in the same time, the resource of time becomes scarce. In economic theory, time is treated as a valuable resource (Mankiw 2004, pp. 4–6); if a resource becomes scarce, its value or price increases (Johnson 1978, p. 53). ‘The increase in the value of time (its increasing scarcity) is felt subjectively as an increase in tempo or pace’ (Johnson 1978, p. 53).

Organizations – especially for-profit organizations – are increasingly required to operate faster, including their communication processes (Suchanek and Entschew 2018, p. 221; Child and McGrath 2001, pp. 1136–1139; Eisenhardt 1989). Digital communication enables this intended acceleration. Digital communication encompasses the conversion of analogue information into digital information and the transmission and processing of digital information. Information is understood as messages received and understood. In this article, I mainly refer to communication via email, instant messaging services, social media channels or other online services which enable digital correspondence based on tablets, smartphones, wearables and the like. Digitizing human interaction through online services and digital communication devices has had a tremendous impact on the way how humans communicate (Royakkers et al. 2018, p. 128). A hyperconnected reality is created, whereby human life is intertwined with such services and devices (Floridi 2015).

Thanks to these services and devices, organizational members in different locations around the world can exchange information at the speed of light extending fact-to-face interactions to distant interactions (Coeckelbergh 2011, p. 85; Tapscott 2014, p. 138; Wright 2011, p. 207). No space needs to be bridged anymore in order to exchange information, as is the case when using letters. Hence, digital communication enables increasing independence of space of the interacting organizational members resulting in time savings (Rosa 2014, pp. 126–127). It also leads to an increase in the frequency and quantity of possible contacts (Coeckelbergh 2011, p. 85). As a result, the acceleration of communication (Brynjolfsson and McAfee 2014, p. 41; Moore 1965, p. 115) leads to an increasing amount of information (Child and McGrath 2001, p. 1136; Zuboff 1988, p. 348).

In general, acceleration describes a growing quantity per time unit; here the quantity refers to communicated amounts of information (e.g., info/ d ↑) (Rosa 2014, pp. 113–118). In organizations, activities like communication are often optimized in order to release time resources. These time resources are often used ‘for more productive activities’ (Chui et al. 2012, p. 11). Productivity of one’s work is understood as output quantity per invested time unit, for instance, more communicated information per day or more services per year. Increasing productivity implies growth.

Indeed, the logic of acceleration in order to be more productive, efficient and grow is nothing new, but rather a functional principle of economic thinking since centuries (Hassan 2003, p. 234). ‘Capitalism and speed are indissolubly connected’ (Hassan 2003, p. 234). If functional principles of classical economic and business theories are the only factors influencing management decisions, reductionist and immoral decisions are highly probable. This will become clearer after understanding the development of the acceleration cycle in organizations and related problems.

## The Acceleration Cycle and a Lack of Time

### Technological Acceleration: Communication Technologies

Since digital devices entered the life of many humans, processes are increasingly accelerating. Moore (1965, p. 115) predicted the exponential growth of the performance of digital devices and thereby, technological acceleration. Innovative communication technologies have been particularly relevant in facilitating this intentional acceleration. This relates to hardware innovations like smartphones, tablets or wearables^2^ as well as to internet-based services like email, instant messaging (e.g., Skype or Slack) or social media channels. Technological acceleration enables humans to save time, which can then be used for more productive activities.

For instance, time savings are created through communicating via email instead of letters. Further time savings are enabled through the optimization of one-to-one (or one-to-many) communication to many-to-many communication (Chui et al. 2012, pp. 10–11). Via email (one-to-one or one-to-many communication), time needs to be invested in order to select all relevant addressees or to formulate the right courtesies. Via Slack (many-to-many communication) preset target groups and different channels are used in order to instantly chat. Slack enables informal interactions by focusing on the shared content and on problem solving. As such, faster communication is facilitated.

### Acceleration of Social Change: Change of Communication Norms

Acceleration of social change means that the present is shrinking (Rosa 2014, p. 133). The present is understood as the time unit of stable expectations and decisions (Rosa 2014, p. 134; Luhmann 1990, pp. 135–136). As a result, social expectations are changing faster. Changing expectations imply uncertainty and insecurity as well as a lack of reliability (Suchanek and Entschew 2018, p. 228). The validity of organizational norms and action patterns would expire faster and expectations would need to be adapted at increasingly shorter intervals (Rosa 2014, p. 249).

**Fig. 1:**
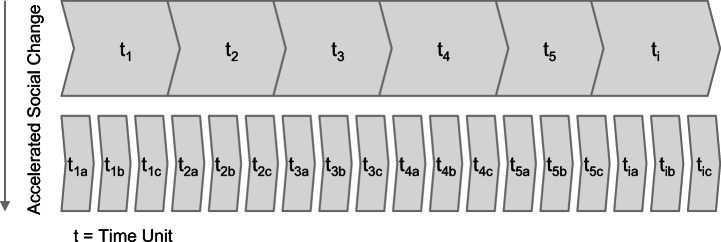
Reduction of Time Units (Source: Own illustration based on Luhmann 1990, pp. 135–136; Rosa 2014, pp. 133–134)

Social change is often induced through new technologies. For instance, while important content in an organization is mainly shared via emails in t_1a_ it may now be mainly shared via Slack or social media in t_1b_ (cf. Fig. 1) creating adapted expectations with regard to communication (e.g., faster responses). This accelerated change of communication norms implies unstable expectations regarding adequate response times, ways of expressing information or channels of communication.

### Acceleration of the Pace of Life: Speed of Communication

**Fig. 2:**
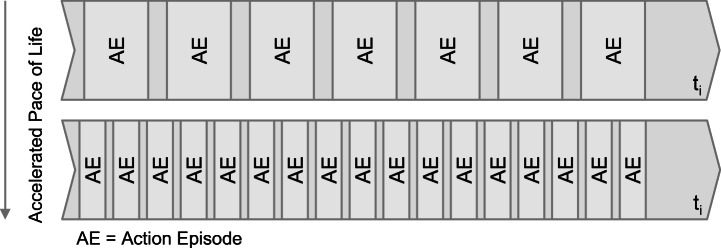
Increase of Action Episodes per Time Unit (Source: Own illustration based on Rosa 2014, pp. 135–137)

The third form of acceleration refers to an increase of action episodes (Rosa 2014, pp. 135–137) (e.g., writing more emails) per time unit (cf. Fig. 2). According to Southerton (2003, p. 12), time is squeezed in order to be utilized and not be wasted.

This increase of action episodes can be realized through:

- a direct increase of the speed of an action e.g., eating faster, reduction of sleep or communicating faster;
- a reduction of breaks, idle times or interims, e.g., an increasing number of organizations allows working, doing training, eating, doing laundry, relaxing, sleeping, etc. within the organization or at home, thereby shortening interims;
- the simultaneous execution of more activities, e.g., multi-tasking: working, eating and commuting simultaneously or communicating via multiple channels with different colleagues.

The accelerated pace of life through increasing communication speed (e.g., writing more emails) leads to higher volumes of communicated information (Rosa 2014, pp. 113–118). Growing information per time unit implies that either more information is communicated in the same time period, or the same quantity of information is communicated in less time: communicated information (q)/ time unit (t) ↑.

In fact, the number of received organizational emails per day has grown by approximately 91% from 2011 to 2018 in Germany (German Association for Information Technology, Telecommunications and New Media 2019). Also, it is expected that from 2019 to 2023 the number of sent and received emails per day will further increase worldwide (The Radicati Group 2019).

### The Accelerating Communication Cycle: A Systematic Lack of Time

The three forms of acceleration through digital communication influencing the perception of time give relevant insights about the ‘time pressure’ paradox based on the difference between collective and individual rationality. According to Rosa (2003, p. 9), an accelerated pace of life possibly influences the ‘experience of time: it will cause people to consider time as scarce, to feel hurried and under time pressure and stress’. Luhmann (1977, p. 46) identifies that also accelerated social change may lead to the feeling of an increasing lack of time since new expectations have to be formed at an increasing pace.

As Rosa (2003, p. 10) points out: ‘If free time decreases in spite of technological acceleration, the only possible explanation is that the quantity of activity itself […] has risen faster than the corresponding technological rate of acceleration’. In other words, abundant time^3^ is created if the rate of technological acceleration is higher than the growth rate (growth may involve an increasing number of information, etc.) (Rosa 2003, p. 10):

1. Abundance of Time:

$$ \mathrm{growth}\ \mathrm{rates}<\mathrm{technological}\ \mathrm{acceleration}\ \mathrm{rates} $$

2. Lack of Time:

$$ \mathrm{growth}\ \mathrm{rates}>\mathrm{technological}\ \mathrm{acceleration}\ \mathrm{rates} $$

If abundant time is directly converted into more activities (e.g., processing more information/ emails) due to more projects/ tasks instead of investing time in existing activities, a lack of time develops (Johnson 1978, p. 55; Rosa 2003, p. 11). The study of Ulferts et al. (2013) gives evidence that demands at work are accelerating thereby organizational members are often required to handle more activities. As mentioned earlier, the logic of acceleration in order to be more productive, efficient and grow is a functional principle of classical economic theory. Following individual rationality, organizational members can react via accelerating the pace of life: More activities are squeezed into one time unit by accelerating the speed of communication, (e.g., writing faster).

However, these techniques are naturally limited (Rosa 2014, p. 244). The resource of time cannot grow; it can only be optimized. Only 24 h per day, 7 days in a week and so on can be optimized. Hence, if the only reaction to growth requirements in organizations is an accelerated pace of life on the micro-level, time would become scarce.

**Fig. 3:**
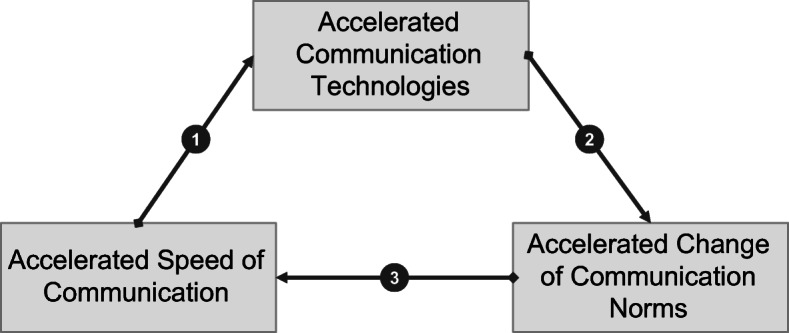
The Accelerating Communication Cycle (Source: Own illustration based on Rosa 2003, p. 12)

This problem could be approached by technological acceleration (Rosa 2014, p. 244): Accelerated communication technologies enable faster exchange of information between organizational members from different locations, for instance, communication via email, as compared to a conventional letter (for the following cf. Rosa 2014, pp. 118–120). Writing and sending an email takes approximately only half of the time (½ t) that a conventional letter would do. That would imply that organizational members are able to communicate twice as much in the same time (q/ ½ t = 2q/ t) (cf. first arrow in Fig. 3). Communicating faster (accelerated speed of communication) and using improved, accelerated communication technologies to further optimize communication are attempts reflecting the alleged solution to a lack of time following individual rationality. However, this reflects the micro-level and neglects the further organizational processes on the meso-level including organizational norms.

Technological acceleration often leads to new norms and action patterns like faster response times, new ways of expressing information or new channels of communication. Hence, without any countermeasures, accelerated communication technologies drive an accelerated change of communication norms (Rosa 2014, p. 248) (cf. second arrow in Fig. 3).

Frequently changing norms often require organizational members to adapt faster to new expectations (e.g., shorter response times). If that holds true, there is pressure in order to keep up with the accelerated change by accelerating the speed of communication (e.g., by writing faster). In other words, the accelerated change of communication norms encourages an accelerated speed of communication (Rosa 2014, p. 250) (cf. third arrow in Fig. 3).

Further, an answer being available on the same day (email) instead of within 2 days (letter) triggers an impulse of answering as soon as possible. This would be accelerated further if digital communication tends to take place in real time by chatting directly (via Slack). If an addressee creates a bottleneck while taking too long to reply, one can easily ‘ping’^4^ them via Slack as a direct reminder in order to accelerate receiving an answer. This creates another force of answering as soon as possible (accelerated speed of communication).

If we now assume that the number of processed emails is four times higher than the number of written letters (4q); or that the number of processed Slack messages is four times higher than the amount of processed messages via email (accelerated speed of communication); the net time consumption for correspondence has increased by 100%, since time would only be saved by a factor of two by technological acceleration (2q/ t < 4q/ t). This phenomenon can be referred to as a rebound effect: Time savings through increasing efficiency are overcompensated and negated through growth effects (more information) of changing human behavior (Berkhout et al. 2000, p. 425; Binswanger 2001, p. 120).

It is often assumed that an increase of efficiency through technological progress by 1 % also leads to a decrease in resource use by 1 % (Binswanger 2001, p. 120). In fact, the reduction in resource use is often below 1 %. Sometimes resource use even increases based on behavioral responses (Binswanger 2001, p. 120).

In the illustration above, the intended efficiency gains through time savings are overcompensated by an increase in information (behavioral response). In the organizational context, economic forces fuel this behavioral response promoting growth. If accelerated communication technologies allow for time savings by a lower rate than the rate of the actual increase of communicated information, a lack of time systematically develops through the cycle of accelerated communication:

$$ \mathrm{growth}\ \mathrm{rate}>\mathrm{technological}\ \mathrm{acceleration}\ \mathrm{rate} $$

This reflects the identified problem: The intention of accelerated communication is saving time, but the desired time reduction is overcompensated if the communicated amount increases at a higher rate (growth rate through accelerated speed of communication > acceleration rate through accelerated communication technologies). If these growth rates are systematically exceeding acceleration rates in organizations – based on missing organizational measures for sustainably managing growth requirements – digital communication systematically leads to the experience of a lack of time.

The call for time saving technologies possibly increases in light of the perceived lack of time due to the limits of the technique of accelerating the speed of communication (Rosa 2014, pp. 250–251) (cf. first arrow in Fig. 3). If accelerated communication technologies (micro-level) are the strategic answer to this problem; the self-reinforcing acceleration cycle (meso-level) is complete.

The dynamics of the communication cycle show that the technologies, which enable the accelerated communication possibly lead to an accelerated change of communication norms and speed of communication calling for further time saving technologies (Rosa 2014, pp. 292–293). If that is the case, (time saving) technologies create their own necessity.

This connection weakens the aforementioned quotation of Martin (2013, p. 33) where he claims that ‘our tools are way superior to what they have ever been’ as a possible solution to increasing acceleration. Indeed, technological tools improve in the course of time, but they are part of the problem. The preceding analysis shows that the technological tools support dealing with a faster pace; at the same time, they reinforce the faster pace. While Martin (2013, p. 33) gives some indication on the phenomenon of an acceleration cycle by mentioning an ‘arms race’ he is not giving enough attention to the consequences of such an ‘arms race’.

The acceleration cycle elucidates the difference between individual and collective rationality (Rosa 2014, pp. 251–252): On the micro-level, faster communication technologies seem to be a plausible solution for the problem of a lack of time. On the meso-level, these technologies are an element of the problem being a stimulus driving the acceleration cycle. The acceleration cycle attempts to explain why organizational members may experience a lack of time, although technologies are saving time. The analysis showed that leadership at the organizational level (meso-level) is needed, rather than simply being left to individual employees seeking personal solutions on the micro-level.

The next section is dedicated to discussing the particular risk of a lack of time when being left to individual employees. A systematic lack of time may lead to an expectation of permanent availability as an excessive expectation potentially harming organizational members. If adequate measures^5^ are not taken, conditions are created which are not conducive for a sustainable work culture.

## Permanent Availability: A Futile Attempt to Cope with a Lack of Time

A systematic lack of time would encourage organizational members to comply with the acceleration pressure. The dynamics of accelerated communication at work potentially lead to an implicit availability demand (Entschew and Suchanek 2017, pp. 354–355; Menz 2017, p. 22). This is further driven by the possibility of communicating any time and everywhere (Boswell and Olson-Buchanan 2016, p. 593; Fenner and Renn 2004, p. 184). As a result, organizational communication processes tend to take place more often (Hassan 2010, p. 369; Rosa 2014, pp. 303–304).

Increasing availability is one way to cope with the acceleration demands on the micro-level. It leads to an accelerated speed of communication, since the volume of communicated information per day increases (Hassan 2010, pp. 371–372; Luhmann 2004, p. 254). Organizational members aim at self- optimization and time savings by using accelerated communication technologies. Both present an alleged solution to a lack of time on the micro-level neglecting the organizational acceleration process on the meso-level.

Self-optimization practices are individual attempts adhering to the availability and acceleration demands. Self-optimization or self-improvement means the permanent improvement of a human’s (opportunities for) actions in order to get the most efficient outcome, like communicating faster (Brinkmann 2017; Cederström and Spicer 2017). The concept of the enterprising self by Rose (1996) is very similar to the self-optimized human. Humans are living by making a project of themselves: they are to work on every part of their life (emotions, employment, health, skills, sexual pleasures, etc.) in order to maximize the worth of their existence to themselves (Rose 1996). This way of life is controlled by an economic mindset including quantifying human actions by collection data on emotions, employment, health, etc.. However, a strong focus on measurable data does not tell everything about human beings missing out important narrative factors (Han 2017).

The development of self-optimization or the enterprising self reflects a strong economic logic excluding everything that is not capable of being measured like perspectives from liberal arts and humanities including morality or emotional intelligence. Morality and skills like emotional intelligence and reflection competence are not an integral part of classical business school academia. This is also the major criticism of Ghoshal (2005) accusing business school academia inducing negative management behavior due to bad theories. Part of the problem is that management theories are mainly following causal or functional modes of explanation. But morality is a social phenomenon. Following Ghoshal (2005), in order to make business studies a science, the role of morality had to be explicitly excluded. Berlin (2002) is in line with this criticism, condemning the approach of reducing human behavior to impersonal social forces. This results in an exclusion of everything that is not directly measurable leading to absurdities in theory and dehumanization in practice like pushing organizational members to be available permanently in order to keep up with excessive acceleration demands.

In summarizing classical economic and management theories, Ghoshal (2005) derives the picture of managers: ‘Combine agency theory with transaction costs economics, add standard versions of game theory and negotiating analysis, and the picture of the manager that emerges is one that is now very familiar in practice: the ruthlessly hard-driving, strictly top-down, command-and-control focused, shareholder-value-obsessed, win-at-any-cost business leader’ (Ghoshal 2005, p. 85). As mentioned at the beginning of this article, managers are often viewed as self-interested and opportunistic maximizers in society. According to Ghoshal (2005) assumptions of business theories influenced the development of the negative management role in society as a process of a self-fulfilling prophecy. Further concepts confirm this negative role of management and associated institutions. One such concept is called greedy institutions. Coser (1974) calls institutions – that request undivided commitment – greedy institutions. Greedy institutions do not consider sustainable levels of organizational members’ energy, but rather exploit their organizational members given that regulations for sustainably managing acceleration and growth requirements are non-existent. Greedy institutions are defined as institutions that ‘seek exclusive and undivided loyalty and they attempt to reduce claims of competing roles and status positions on those they wish to encompass within their boundaries. Their demands on the person are omnivorous’ (cf. Coser 1974, p. 4). They are based on voluntariness instead of external coercion, in contrast to ‘total institutions’ like prisons. Greedy institutions rely on commitment and loyalty by their members (cf. Coser 1974). Membership is highly desirable and ties to other institutions like family or communities are weakened based on the encompassing claims by the institution (cf. Coser 1974, p. 6). The sociologist Marianne Egger de Campo (2013, p. 969) affirms this with her contemporary interpretation of Coser’s work. She asserts that greedy institutions unlimitedly claim all parts of an individual’s energy. Among others, Egger de Campo (2013) characterizes management consultants reflecting traditional business practices and social media as a form of digital communication as greedy institutions. Egger de Campo (2013) as well as Sennett (1998) characterize capitalist organizations as greedy institutions due to the unconditional allegiance and an extended grasp required by management personal and consultants. This can be interpreted as a reflection of the current management education in business schools. If business schools focus their education on shareholder value and view ethics and morality as constraints (Pirson 2017; Amann et al. 2011), organizations are incentivized to foster the encompassing (greedy) utilization of an individual’s commitment and loyalty.

The study of Middleton and Cukier (2006) provides evidence on these ‘greedy’ claims. Organizations implicitly request undivided commitment through an increasing usage of digital media. Organizational members confirm that checking emails is the first thing they do afterwaking up in their beds at home (cf. Middleton and Cukier 2006, p. 256). The study gives insights that digital devices are more and more used at any time for work-relatedpurposes (Middleton and Cukier 2006, pp. 256–257): ‘Users [of digital media] were strongly committed to their patterns of use, showing evidence of fixation and compulsion. They used their devices on bicycles, in cars, airplanes,trains, taxis and buses, at all times of day and night. They used them while on vacation (rationalizing [sic] this behavior [sic] as a way of staying in touch) and took them to the beach and to the park. They used them in meetings, while on the telephone and when watching television. When the device ‘buzzed’, many users could not resist the urge to immediately check the new email message it signalled.’

While there is evidence of a feeling of compulsion to be available permanently, organizational members are not externally forced to show this behavior. It is based on voluntariness. Facing work cultures of greedy institutions, the feeling of a lack of time intensifies because undivided commitment is not leaving time for other commitments like family responsibilities or community work (Rosa 2014, pp. 304–305; Coser 1974, p. 8).

If no routines, no training and no conducive work culture exist, the risk of ‘overcommitting’ to one’s organizational role prevails (Sullivan 2014, 1, 7). There may be cases of ‘undercommitting’ which are relevant for organizations too but this goes beyond the scope of this paper. Sullivan (2014, p. 7) points out that adequate commitment is especially dependent on work culture, since it is often the case that policies and rules get neutralized by strong (greedy) cultures. Reasons for ‘overcommitment’ are manifold: increased business, pressing deadlines, etc.

In some industries, it is well known that the job demands to work very long hours and to sacrifice time for family and social pleasures for work (Sennett 2012, p. 149). Nevertheless, its overarching scope through a lack of time due to accelerated digital communication makes it an even more common phenomenon, thereby increasing the risk of immoral behavior including managers having excessive expectations regarding organizational members’ availability.

The major problem is that organizational members may lose control of the situation as soon as permanent availability develops into a clear expectation (change of communication norms). Also, organizational members – who more or less freely chose to be available permanently – might experience a loss of control at some point in time.

Schwartz (2007, p. 104) identifies two explanations for this. Firstly, expectations may rise ‘excessively’ (growing without any threshold): ‘Like the mechanical rabbit at the dog-racing track that speeds along just ahead of the dogs no matter how fast they run, aspirations and expectations […] speed ahead of their realization, no matter how liberating the realization becomes.’

Secondly, communication may increase in a way that organizational members feel overwhelmed and not able to cope with it (Schwartz 2007, p. 104). This may refer to deciding between adequate action patterns (change of communication norms) for instance, ‘Is availability required during weekends’? or ‘Should the email account be checked after the end of the work day’? In general, ‘What is the right thing to do’? But, this may also refer to the quantitative increase of information (accelerated speed of communication) encouraging organizational members who are permanently available to communicate and work more.

A study on availability showed that permanently available organizational members in Germany are working more overtime hours: While permanently available employees work 6 overtime hours, employees of the control group are working 1.35 overtime hours (Hassler and Rau 2016, p. 30). Also, the perceived workload is higher and the perception of having control is lower for permanently available employees compared to the control group (Hassler and Rau 2016, p. 31).

In another study of Hassler et al. (2016, p. 33), the increased amount of overtime hours is confirmed: While permanently available employees work 7.4 overtime hours, employees of the control group are working only 2.6 overtime hours.

Seemingly, permanent availability may function as a coping mechanism for a lack of time in the short term. However, it only copes with the symptoms of the problem (increasing information) instead of coping with a root cause on the meso-level (accelerating communication cycle). The constant interplay of the micro- and meso-level leading to the problem of a perceived lack of time has become clear throughout the analysis. As indicated several times, the mindset of managers is bred in business schools which - in many cases - still rely on a negative, homogenous and reductionist perspective on the human nature. Hence, the analysis aims at functioning as a call for business schools and managers to extend their theories and thinking to interdisciplinary fields realizing a heterogenous view on human beings including moral values and other important immeasurable phenomena. Hence, ‘[...] it is all the more important that not only firms, but also their stakeholders, invest in the ability of moral discernment as the most elementary prerequisite for sustainable social cooperation for mutual advantage’ (Suchanek 2017, p. 88). A specific attempt is promoted by Litton and Wacker (2020) calling for paired courses combining business studies with liberal arts in order to equip future business leaders with important skills. These skills do not only relate to classical economic/ management theory focusing on measurable outcomes, but also on immeasurable, highly important skills including communication, emotional intelligence or reflection competence; all of which are relying on a heterogenous perspective of the world. There are already some business schools trying to follow such attempts. But these are still exceptions which need to be taken seriously and which need to be developed to mainstream business studies. 

## Conclusion and Implications for Practice and Research

This paper revolved around the thesis that digital communication leads to the experience of a lack of time if an accelerating communication cycle prevails. First, the analysis showed that digital, accelerated communication leads to time savings and that communication technologies support organizational members dealing with acceleration (micro-level). However, it was also shown that these technologies may induce behavioral responses leading to an increase in communicated information overcompensating the time savings. Hence, digital communication technologies only present a short-term solution. They are even part of the problem, since they push an acceleration cycle forward (meso-level) leading to the experience of a lack of time.

Identifying and responsibly dealing with a lack of time would require a sufficient understanding of one’s (digital) environment. An inherent part of the environment to be understood is acceleration. In case the problem of an acceleration cycle is identified, there needs to be the intention as well as the ability to sustainably cope with it on the organizational level. This is especially relevant in academia with a focus on business schools and in practice with a focus on managers. Business schools are framing the context for organizations. It is the responsibility of business schools to rework the strong focus on measurable elements towards a more heterogenous view of humans and the world (of organizations). Since organizations are embedded in a world with high demands regarding growth and productivity, it is the responsibility of managers to protect organizational members from requirements like permanent availability. Technologies need to be used sustainably and conscious decisions regarding where to draw the line need to be made, instead of allowing self-reinforcing acceleration processes determining where to (not) draw the line.

Future research should focus on this responsibility to protect organizational members from excessive requirements in accelerated times. Further, other measures for sustainably dealing with acceleration need to be identified. Finally, the development of a lack of time based on an acceleration cycle needs empirical verifications.

## Footnotes

1 The term organizational members includes managers and employees at non-managerial level.

2 Wearables are smart-digital devices that enable written and voice communications as well as continuous monitoring and alerts on personal (health) data while worn on or in the body (Tapscott 2014, pp. 123–124).

3 In organizations, abundant time refers to freedom of action in which organizational members have time for social activities (e.g., analogously or digitally chatting with each other regarding self-chosen content). It also refers to possible time slots used to step back from one’s current role or situation for reflecting. In times of permanent connectivity, it also means actual free time used privately.

4 Pinging someone means to get a person’s attention by sending a digital signal directly to this person’s digital account on a platform. Hence, it is understood as a tool of quick communication.

5 An adequate measure relates to the responsibility of managers. Simons (2005, pp. 157–158) is pointing out the responsibility of managers to protect employees e.g., from excessively using the possibility of a home office, instead of having excessive expectations.
